# Trophoblast Stem-Cell-Derived Exosomes Alleviate Cardiotoxicity of Doxorubicin via Improving Mfn2-Mediated Mitochondrial Fusion

**DOI:** 10.1007/s12012-022-09774-2

**Published:** 2023-01-07

**Authors:** Junfeng Duan, Xiaoli Liu, Song Shen, Xi Tan, Yi Wang, Lian Wang, Lina Kang, Kun Wang, Zhonghai Wei, Yu Qi, Lei Hu, Biao Xu, Rong Gu

**Affiliations:** 1 State Key Laboratory of Pharmaceutical Biotechnology Department of Cardiology, Medical School of Nanjing University, Nanjing Drum Tower Hospital, No. 321 Zhongshan Road, Nanjing, 210008 China; 2 State Key Laboratory of Pharmaceutical Biotechnology, Department of Cardiology, Nanjing Drum Tower Hospital, Nanjing Drum Tower Hospital Clinical College of Nanjing University of Chinese Medicine, No. 321 Zhongshan Road, Nanjing, 210008 China

**Keywords:** Exosomes, Trophoblasts, Mitochondrial fusion, Doxorubicin, Cardiotoxicity

## Abstract

Doxorubicin (Dox) is an anticancer drug widely used in tumor chemotherapy, but it has the side-effect of cardiotoxicity, which is closely related to mitochondrial damage. Mitochondrial dynamics is a quality control mechanism that usually helps to maintain a healthy mitochondrial pool. Trophoblast stem cell-derived exosomes (TSC-Exos) have been shown to protect cardiomyocytes from DOX-induced cardiotoxicity. To explore whether the cardioprotective role is mediated by the regulation of mitochondrial dynamic mechanism, TSC-Exos were isolated from human trophoblast stem cells by ultracentrifugation and characterized by Western blot and transmission electron microscopy. Cellular experiments of H9c2 cardiomyocytes co-cultured with Dox and TSC-Exos were performed in vitro to determine the levels of reactive oxygen species generation and apoptosis level. An animal model of heart failure was established by intraperitoneal injection of Dox in vivo, therapy mice were received additional intracardiac injection of TSC-Exos, then, the cardiac function, cardiomyocyte apoptosis and mitochondrial fragmentation were ameliorated. Histology assays suggest that Dox caused an increased tendency of mitochondrial fission, which was manifested by a decrease in the average size of mitochondria. By receiving TSC-Exos treatment, this effect was eliminated. In summary, these results suggest that TSC-Exos alleviate DOX-induced cardiotoxicity through antiapoptotic effect and improving mitochondrial fusion with an increase in Mfn2 expression. This study is the first to provide a potential new treatment scheme for the treatment of heart failure from the perspective of the relationship between TSC-Exos and mitochondrial dynamics.

## Introduction

Doxorubicin (Dox) has been a commonly utilized chemotherapeutic drug in cancer chemotherapy for over 100 years [1]. However, cardiotoxicity has always been an unavoidable toxic side effect with long-term usage, which can further develop into irreversible myocardial damage, eventually leading to congestive heart failure [2–4]. Dox is significantly concentrated in mitochondria, forming a complex with the elevated iron, leading to the generation of reactive oxygen species (ROS). Some investigations have linked Dox-induced cardiotoxicity to mitochondrial damage [5], characterized by an imbalance in the mitochondrial quality control mechanism and ultimately leads to apoptosis and death [6]. Dox cardiotoxicity is now caused, at least partly, by an increase in mitochondrial fragmentation and faster lysosomal breakdown of mitochondria [7–9].

Significantly, mitochondrial dysfunction is the characteristic of heart failure in patients and animal models [10]. Cellular mitochondria are interconnected, and their morphology is in a dynamic balance of fission and fusion [11]. Together with mitochondrial autophagy, they are collectively referred to as mitochondrial dynamics. Mitochondria use mitochondrial dynamics to adjust to changes in the intracellular environment and sustain their functions. Mitochondrial dynamics suggest that the mitochondria are constantly fissioning and fusing [12]. Mitochondrial dynamics, or the adaptability of mitochondria to cell changes, is an essential mitochondrial quality control mechanism [13, 14].

Exosomes have become increasingly established as a therapeutic tool, and in recent years more and more types of stem cell-secreted exosomes have been used for the treatment of heart failure [15–17]. As a group of stem cells produced in the early stage of cell differentiation in monolayer embryos, human trophoblast stem cells (TSCs) have also been proven to be protective against heart failure [18]. Previous studies have shown that the secreted TSC-Exos can lessen DOX-induced cardiac injury by regulating the expression of miR-200b to play an antiapoptotic and anti-inflammatory role [19]. So far, no one has studied the relationship between exosomes secreted by trophoblast stem cells and mitochondrial dynamics. Therefore, our study explored the influence of TSC-Exos on the mitochondrial dynamics of Dox-induced cardiotoxicity.

## Materials and Methods

### Animals

Mice were C57BL/6 male mice aged 8 weeks. The work was authorized by Nanjing Drum Tower Hospital’s Institutional Ethics Committee, and all animal experiments were performed in accordance with the Guide for the Care and Use of Laboratory Animals (8th edition). All mice were purchased from the Model Animal Research Center of Nanjing University, placed in standard laboratory cages, fed standard laboratory chow, and maintained in a suitable temperature and light environment. All animals were sedated with isoflurane by 1.5% inhalation at the end of the experiment, followed by euthanasia by cervical dislocation. Dox (5 mg/kg; PHR1789; Sigma) was given intraperitoneally to mice in the DOX group once a week for four weeks. Mice in the treatment group got an intracardiac injection of an additional 25 μl of PBS containing 50 μg TSC-Exos. All groups had intramyocardial injections with thoracotomy at three distinct points.

### Isolation and Characterization of Exosomes

Under normal culture conditions (37 °C, 5% CO2), TSCs (HTR-8/Svneo; ScienceCell; USA) were grown in DMEM supplemented with 10% FBS and 1% penicillin (Invitrogen; USA) solution. After the cells fused to 70% to 80%, they were cultured for another 48 h in DMEM with 5% exosome-depleted fetal bovine serum (Cat: C38010050; ViVaCell; Shanghai). The medium was centrifuged at 300×*g* for 10 min to remove cells, then 2000×*g* for 20 min to remove cellular debris for exosome isolation. After that, the supernatant was centrifuged at 10,000×*g* for 30 min to remove microvesicles. Finally, exosomes were extracted after 60 min of ultracentrifugation at 100,000×*g*. Exosomes are resuspended in PBS, a small portion is used for immediate experiments, and the rest is stored at − 80 °C [20]. After gradient alcohol dehydration and permeation with varied ratios of acetone and epoxy resin solutions for 48 h, the cell samples were subsequently embedded in epoxy resin. After that, 3 percent uranyl acetate and lead citrate were used to stain the sections of the cut-embedded samples. We observed the morphological structure of exosomes with transmission electron microscopy and measured their size distribution with nanoparticle tracking analysis (NTA). The Strokes-Einstein equation was used to calculate the size and concentration of particles using the NTA 2.3 program, which tracked and analyzed particles from 10 to 2000 nm. Exosome surface marker proteins were detected using Western blotting (CD63, TSG101, and CD9).

### Cardiomyocyte Culture and Treatments

Procell (Wuhan, China) provided the H9c2 cardiac myoblast cells, which were grown in DMEM (Gibco, USA) with 10% fetal bovine serum and 1% 100 U/mL antimicrobial penicillin. Cells were divided into the control group, the Dox group, and the Dox + Exo group. Cells were grown in an incubator maintained at 5% CO2 and 37 °C, with regular fluid changes, and passaged when the cells had grown to more than 70% of the bottom area of the culture flask. Dox (1 µM) was given in co-culture to the Dox group and the Dox + Exo group for 24 h. In addition, the Dox + Exo group received 20 µg of exosomes.

### Echocardiography

Transthoracic echocardiography was used to assess the cardiac function of the mice. Mice were first anesthetized with 1.5% isoflurane, and their respiratory rate and body temperature were continuously monitored. Then, the 2-dimensional M-shaped images were obtained on short and long-axis views of the papillary muscle measured over 3 consecutive cardiac cycles. The parameters of left ventricular end-diastolic internal diameter (LVDd), left ventricular end-systolic internal diameter (LVDs) were measured separately using a double-blind method, and left ventricular ejection fraction (EF) and left ventricular short-axis shortening (FS) were calculated.

### Western Blotting

RIPA buffer was used to homogenize and lyse the heart tissues, exosomes, and H9C2 cells (Beyotime; Jiangsu). A sufficient amount of protein was extracted from myocardial tissue and cardiomyocytes using RIPA lysate, and then the BCA protein assay kit was used to determine the protein concentration (Thermo; USA). Proteins were separated in SDS-PAGE and then transferred to PVDF membranes. After blocking with 5% skim milk, the membranes were subsequently incubated overnight at 4 °C with antibodies from Abcam (USA): CD63 (Cat: ab213090), CD9 (Cat: ab92726), TSG101 (Cat: ab125011), Opa1 (Cat: ab157457), Drp1 (Cat: ab184247), Mfn2 (Cat: ab124773), and GAPDH (Cat: ab181602). The membranes were then rinsed three times with TBST before being dripped with the rabbit anti-mouse IgG conjugated to HRP (1:10,000). The enhanced chemiluminescence (ECL) detection kit was used to detect the blots (Beyotime; China).

### Annexin V/PI Staining for Cell Apoptosis

Apoptosis levels of H9c2 cells were detected by Annexin V/PI staining (Cat: KGA108-2; Keygen; China). Briefly, cells were first digested using trypsin and collected at a concentration of approximately 1*10^6 cells/ml. Then, the cells were resuspended in a 200 µl binding buffer and then stained with 5 µl Annexin-FITC and PI. The Flow cytometer (BD; USA) and the FlowJo V10 software were used to analyze and calculate the apoptotic rate.

### Mitochondrial Membrane Potential Detection

JC1 staining was used to examine the mitochondrial membrane potential (Cat: C2006; Beyotime). Cells were firstly rinsed with ice-cold PBS and then stained for 30 min at 37 °C with 2.5 g/mL JC1. Fluorescence microscopy was used to examine the cells after they had been washed with binding buffer. The ratio of relative aggregate to monomer (red/green) fluorescence intensity is displayed.

### Histology Assay

The hearts of mice were removed and placed in 4% paraformaldehyde (ph 7.4) and then embedded in paraffin wax. The sections were stained by Masson staining and HE staining and then histologically observed under a high-resolution microscope (Leica, Japan). And the heart tissue specimens used for electron microscopic observation were first fast fixed with 2.5% glutaraldehyde in 0.1 m sodium phosphate (pH 7.2), washed in the same buffer at 4 °C for 1 h, and fixed with 1% osmium tetroxide in sodium phosphate at 4 °C for 1 h. Each step lasted 10 min starting from 50%, followed by 2 changes of propylene oxide. These tissues were then immersed in Araldite. Ultrathin sections were stained using magnesium uranyl acetate and lead.

### Statistical Analysis

Data from our study were presented as the mean ± Standard Error of Mean (SEM). Statistical differences were assessed using SPSS software and a one-way ANOVA analysis (GraphPad, La Jolla, CA, USA) (IBM SPSS, Chicago, IL, USA). T-tests for unpaired students were employed to compare the two groups. A statistically significant value of P < 0.05 was used.

## Results

### Identification of TSC‑Derived Exosomes

**Fig. 1:**
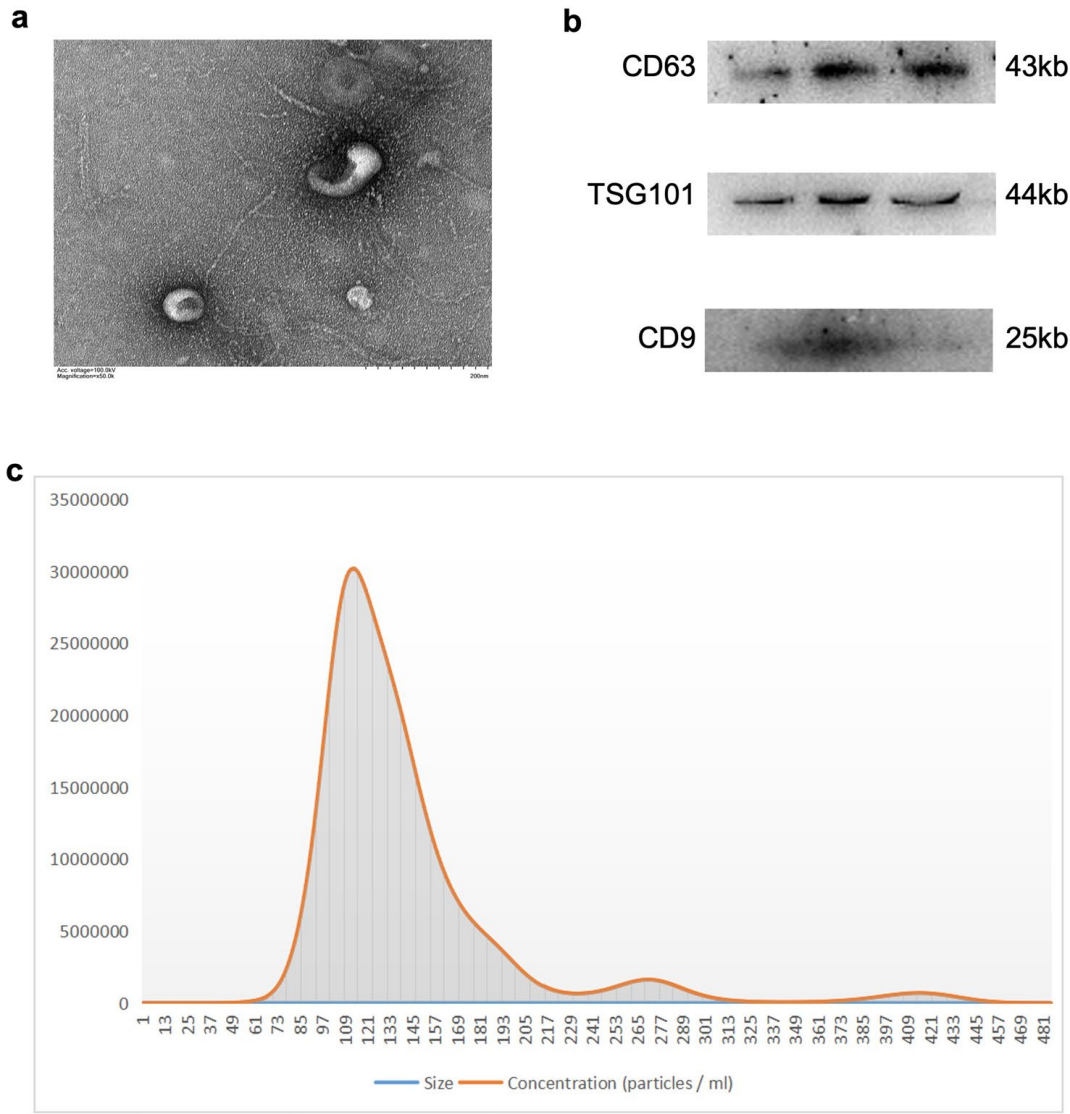
TSC-Exos isolation and characterization. **a** transmission electron microscopy images of exosomes. **b** the expression of exosomal markers (CD63, TSG101and CD9) was confirmed by immunoblotting. **c** the sizes and concentrations of exosomes were measured by NTA

We isolated exosomes from trophoblast stem cells by ultracentrifugation. TSC‑derived exosomes were identified by Western blot and were positive for the exosome markers (CD63, TSG101, and CD9), and particle size analysis showed that the average diameter of exosomes was 113 nm (Fig. 1c).

### TSC‑Exos Improve Cardiac Function in Dox‑Induced Heart Failure

**Fig. 2:**
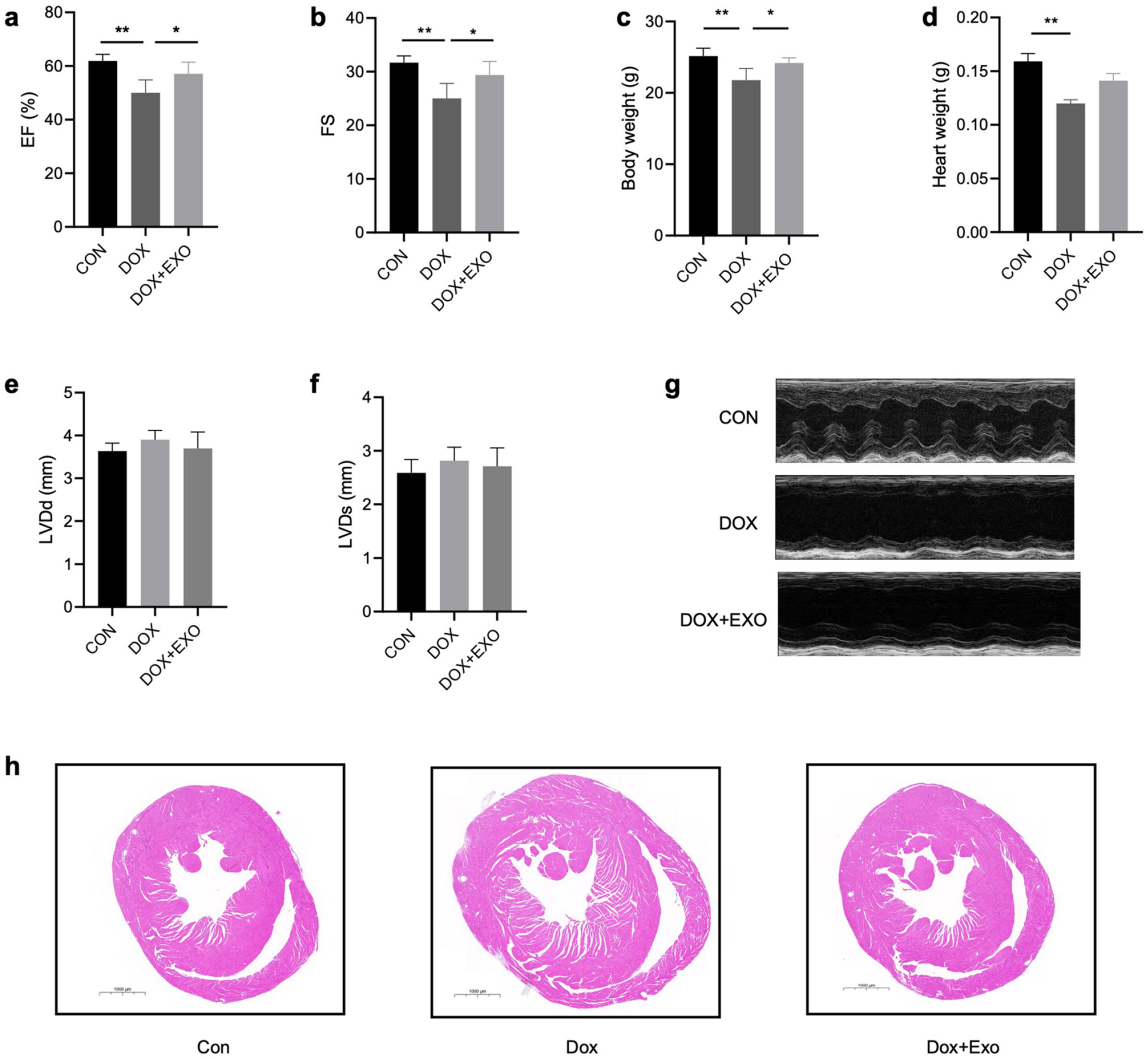
C57BL/6 mice were divided into three groups, control group and Dox group (20 mg/kg for 28 d) given an intraperitoneal injection of Dox for 4 weeks and Dox + Exo group given an additional intracardiac injection of Exo (50 µg), and the cardiac function of each group was assessed by echocardiography after one month. **a** EF; **b** Fractional shortening; **c** Body weight; **d** Heart weight; **e** LVDd; **f** LVDs; **g** Echocardiographic images of each group after 4 weeks; **h** Myocardial tissue of mice using H&E staining. Mean SEM, *n* = 6 (panel a-h), *p < 0.05

After 4 weeks of treatment, we measured the cardiac function of mice by echocardiography. The results showed that compared with the control group, the body weight and heart weight of Dox group mice have significantly reduced (Figs. 2c and d), the cardiac function has deteriorated considerably, and the EF and FS were reduced (*P* < 0.05), but there was no significant difference in LVDd and LVDs. After treatment with exosomes secreted by trophoblast stem cells, the body weight, EF, and FS of mice were significantly up-regulated, while there was no significant difference in heart weight, LVDd, and LVDs (Figs. 2a, b, e and f). Additionally, myocardial tissue sections of mice were stained by hematoxylin–eosin staining as shown in Fig. 2h, the heart tissue in the Dox group had enlarged chambers and thinner ventricular walls than the control group, and this was improved in the Dox + Exo group.

### TSC‑Derived Exosomes Reduce Myocardial Fibrosis and Cardiomyocyte Mitochondrial Fragmentation

**Fig. 3:**
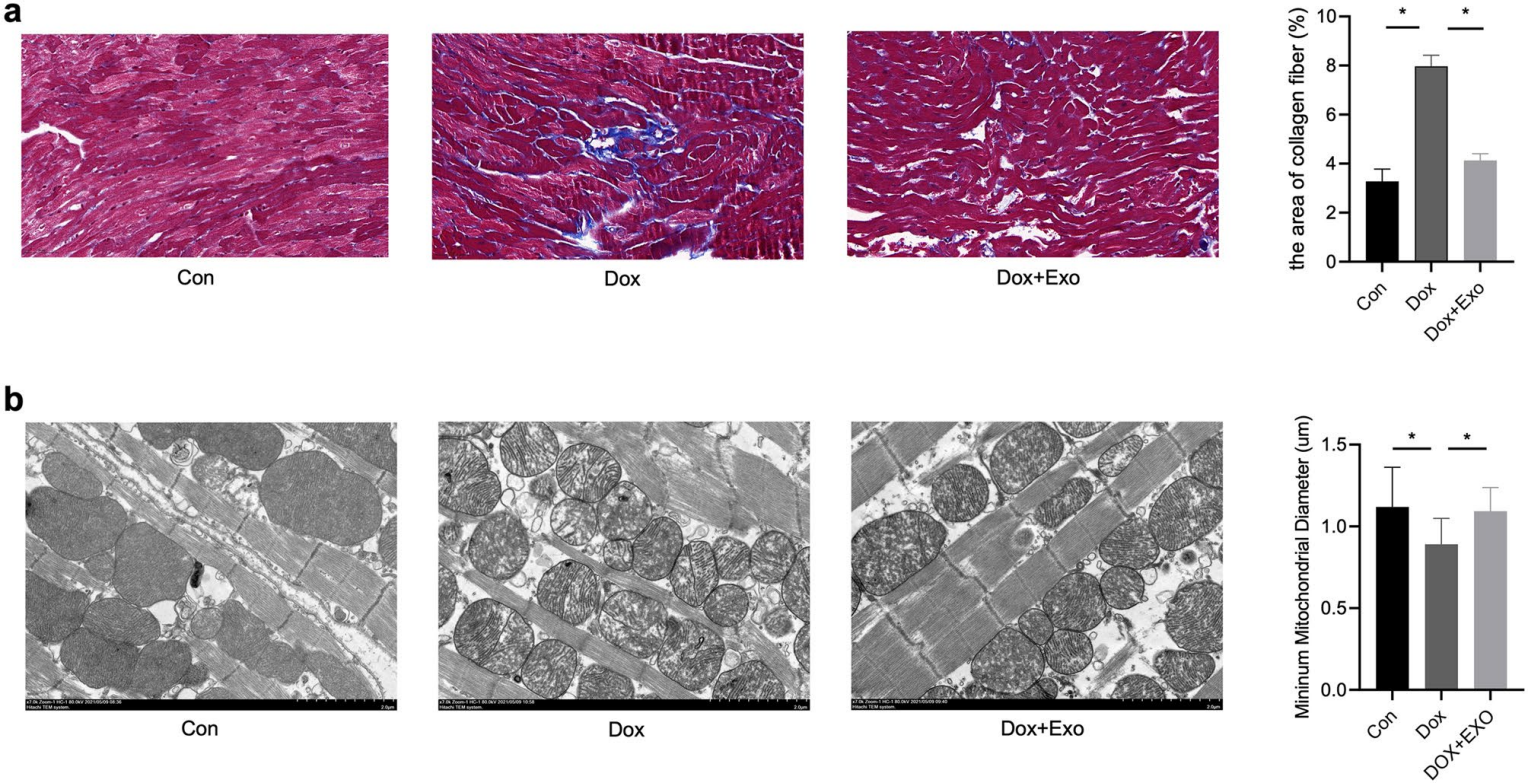
Masson staining of fibrotic regions in distinct groups. **a** exosomes reduce the degree of fibrosis caused by DOX. **b** exosomes attenuated Dox-induced mitochondrial fragmentation. ImageJ was used to examine mitochondrial morphology. The size and number of mitochondria, as well as the major and minor axes, were calculated. Data were analyzed by ANOVA, *n* = 5 (panel a–b). *p < 0.05

Masson and electron microscope stained the myocardial tissue samples of mice. Masson staining showed that the degree of fibrosis in the Dox group was significantly higher than that in the control group, while the degree of fibrosis in Dox model mice treated with exosomes was reduced considerably, indicating that the exosomes secreted by trophoblast stem cells can improve Dox-induced myocardial fibrosis (Fig. 3a). In contrast, the situation in the Dox group was significantly improved after the TSC-Exos intervention. The short-axis diameter of mitochondria was similar to that in the control group, myocardial mitochondria were still slightly damaged, and mitochondria were swollen slightly; the mitochondrial cristae were slightly clear (Fig. 3b). It suggests that the exosomes secreted by trophoblast stem cells can improve the myocardial mitochondrial damage induced by Dox.

### TSC‑Derived Exosomes Attenuated Dox—Induced ROS and Apoptosis Level

**Fig. 4:**
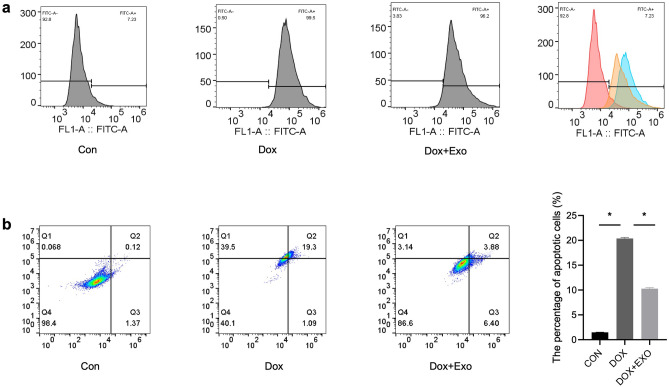
The levels of reactive oxygen species production and apoptosis in H9c2 cardiomyocytes were measured in vitro by flow cytometry. **a** ROS generation levels in each group. **b** apoptosis of cardiomyocytes in each group, *n* = 6 (panel a–b). *p < 0.05

Previous studies have shown that DOX causes myocardial injury by increasing oxidative stress and apoptosis levels. To further investigate TSC-Exos’ effect on it, we used flow cytometry to determine the levels of reactive oxygen species generation and apoptosis in H9c2 cardiomyocytes. The results indicated that the Dox group had a higher level of reactive oxygen species than the control group. In comparison, the treatment group had a lower level than the model group (Fig. 4a). Apoptosis was significantly higher in the Dox group than in the control group. TSC-Exos treatment effectively decreased the apoptosis level of cardiomyocytes (Fig. 4b) (*p* < 0.05).

### TSC‑Derived Exosomes Improve the Mitochondrial Membrane Potential and Attenuate Mitochondrial Fission via Mfn2

**Fig. 5:**
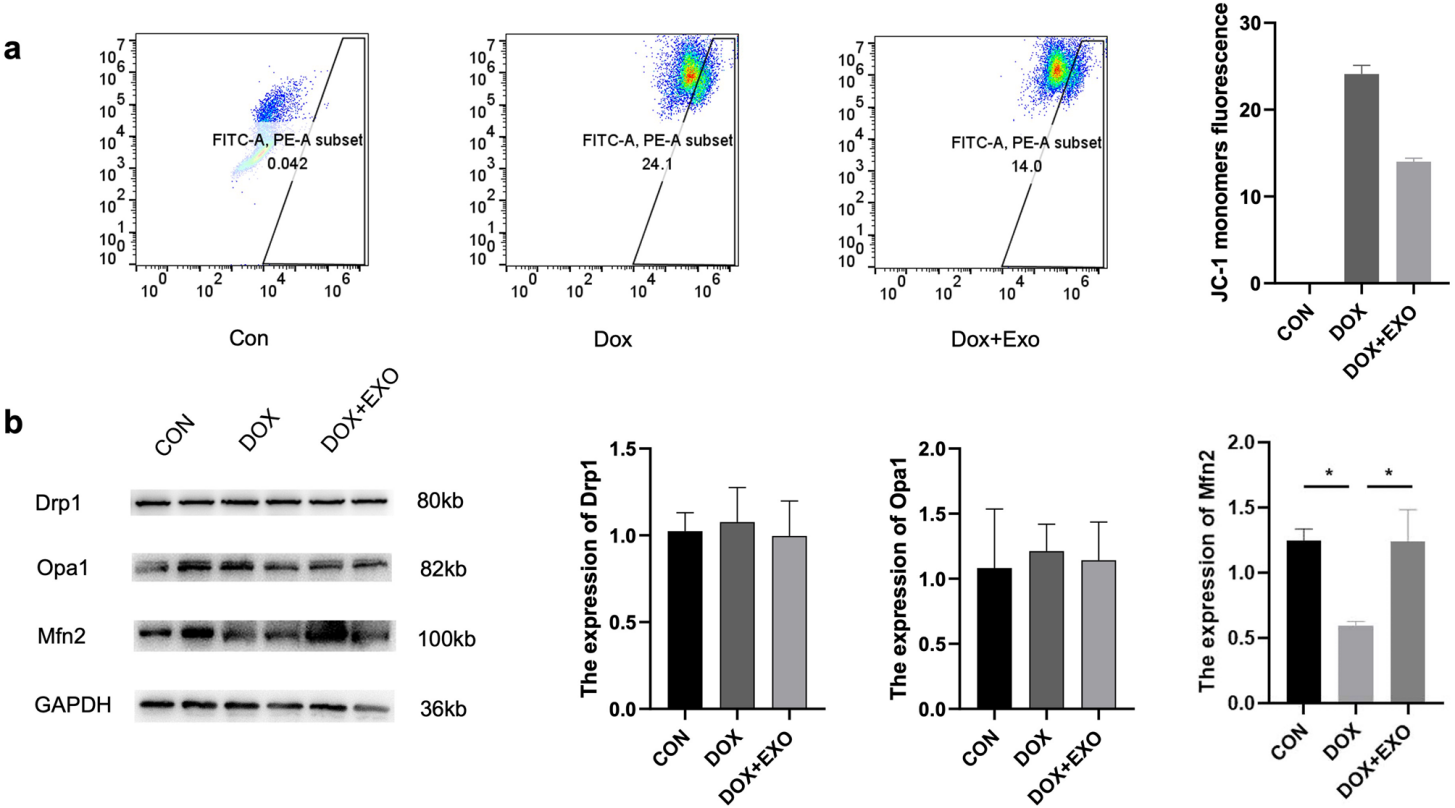
**a** the concentration of JC-1 monomer in each group of cardiomyocytes was measured by flow cytometry and used to reflect the level of mitochondrial membrane potential. **b** the expression and statistical results of mitochondrial morphology-related proteins (DRP1, OPA1, and MFN2) were detected by Western blot. All data are expressed as the mean ± SEM (*n* = 5). *p < 0.05

The fluorescent probe JC-1 monomer fluorescence intensity was used to reflect the level of change in mitochondrial membrane potential in H9c2 cardiomyocytes detected by flow cytometry. The results indicated that the model and treatment groups had greater intensity of JC-1 monomer fluorescence than the control group, implying a decrease in mitochondrial membrane potential. However, the decrease in the treatment group was less than that in the Dox group (Fig. 5a). Drp1, Opa1, and Mfn2 expression levels in myocardial tissues of C57BL/6 mice in each group were determined using the Western blot method. As shown in Fig. 5b, the expression levels of Drp1 and Opa1 in myocardial tissues of C57BL/6 mice were not significantly different, while Mfn2 expression was significantly decreased in the Dox group, and the expression of Mfn2 was effectively increased by receiving TSC-Exos treatment (Fig. 5b).

## Discussion

Dox chelates iron ions and triggers the generation of free oxygen radicals, especially free hydroxyl radicals, resulting in lipid peroxidation of the cardiomyocyte membrane and damage to the myocardial mitochondrial DNA. Mitochondria are essential organelles in cells and are highly distributed in cardiomyocytes. Their functions include generating energy, regulating cell survival (apoptosis), synthesizing ROS, and controlling intracellular calcium concentration. Previous studies have shown that mitochondrial dysfunction after Dox employment is vital in inducing cardiomyocyte apoptosis [20]. DOX accumulates in mitochondria through a variety of non-enzymatic mechanisms, leading to increased oxidative stress and mitochondrial dysfunction. Our study found a significant increase in the level of oxidative stress and the apoptosis of cardiomyocytes after Dox treatment, which is also consistent with findings from previous studies. Numerous studies have shown that mitochondrial dynamics play a unique role in mitochondrial-mediated cell death. Mitochondrial dynamics is defined as the adaptation of the mitochondria to changes in cells. The mitochondria continuously undergo fission and fusion. The imbalance in mitochondrial fission and fusion has been shown to promote sustained mitochondrial rupture, leading to the initiation of mitochondrial apoptosis. Several studies have described the interaction and colocalization of mitochondrial mitotic protein and pro-apoptotic protein (Bax) in mitochondrial mitotic foci, which suggests that mitochondrial dynamics are involved in mitochondrial-mediated cell death, which subsequently activates the pathway to cell death [21].

Mitochondrial fission begins with mitochondrial contraction caused by endoplasmic reticulum tubules. Endoplasmic reticulum tubules orient and determine the fission site, accompanied by mitochondrial fission [22]. Drp-1 is a dynamic protein GTPase protein located in the cytosol. After activation, it is transferred to the fission site on mitochondria for further mitochondrial separation [23]. Mfn2 and Opa1 are important mitochondrial fusion-related proteins [24, 25]. Previous studies have revealed that the conditional deletion of cardiac Mfn2 leads to mitochondrial dysfunction and results in myocardial hypertrophy and dysfunction [26, 27]. Since Mfn2 is responsible for mitochondrial fusion, the lowering of Mfn2 may impair the synergy of fission and fusion dynamics. In a previous study, Mfn2 knockout mice exhibited mitochondrial fragmentation, which resulted in the reduction in the mitochondrial permeability transition pore(MPTP) and aggravated the mitochondrial dysfunction caused by ROS; another study showed that ischemia–reperfusion injury resulted in the low expression of the Mfn2, which resulted in mitochondrial and cardiac dysfunction [28]. There is further evidence that Mfn2-mediated restoration of mitochondrial fusion improved mitochondrial oxidative metabolism in Dox-treated cardiomyocytes, reduced cellular damage, and decreased the level of mitochondria-derived oxidative stress [29]. Therefore, promoting Mfn2-mediated mitochondrial fusion is also a potential strategy to mitigate Dox-induced cardiotoxicity.

Findings from our data indicate that the down-regulation of Mfn2 in the cardiomyocytes of mice treated with Dox promoted the fission of mitochondria. The average short-axis diameter of myocardial mitochondria in the Dox group was significantly shorter than that in the control group. The mitochondria structure was abnormal, arranged disorderly, with a blurred or evenly broken mitochondria crest in the Dox group. However, there was a significant improvement after the TSC-Exos intervention. In the treatment group receiving TSC-Exos therapy, the diameter of the mitochondria short axis was consistent with that of the control group; the myocardial mitochondria were still slightly damaged, with slightly clear mitochondrial cristae. It suggests that the exosomes secreted by the trophoblast stem cells have an inhibitory effect on myocardial mitochondrial damage induced by Dox.

Furthermore, this study showed mitochondrial fission could be aggravated by Dox through the downregulation of Mfn2 expression in cardiomyocytes. In contrast, the expression of Drp1 has no significant change compared with the control group, which is consistent with the previous findings reported [8]. However, this does not affect that Drp1 can be used as a potential therapeutic target. It could be attributed to the relative reduction in mitochondrial fusion, which makes the balance of mitochondrial dynamics more inclined towards fission. Therefore, some previous studies have also demonstrated the feasibility of reducing Dox-associated cardiotoxicity by inhibiting Drp1 expression, which could rebalance the scale between mitochondrial fission and fusion and ameliorate the Dox-induced decline in cardiac function [30]. Moreover, our study revealed that TSC-Exos could reduce the cardiotoxicity of Dox by affecting the mitochondrial dynamics. TSC-Exos enhances mitochondrial fusion by increasing the expression of Mfn2, reduces the increasing trend in mitochondrial fission in cardiomyocytes caused by Dox, rebalances the synergy of mitochondrial dynamics, and reduces the decline in the potential of the mitochondrial membrane and the production of ROS in cardiomyocytes caused by Dox. Previous studies have shown that TSC-Exos inhibits cardiomyocyte apoptosis by up-regulating the antiapoptotic protein Bcl-2 and reducing the expression of miR-200b in cardiomyocytes, our study validated the cardioprotective effect of TSC-Exos on this basis and further revealed that TSC-Exos could improve mitochondrial function by affecting the expression level of Mfn2.

However, the findings of this study have to be seen in light of some limitations. First, this study was limited to the measurement of mitochondrial morphology by tissue electron microscopy and was not further validated at the cellular level. Second, the present study did not further investigate the upstream and downstream molecular mechanisms of Mfn2. Further studies could be performed to explore pathways through which the exosomes secreted by the trophoblast stem cells alter the expression of Mfn2 and the changes in the mitochondrial dynamics. Third, reported study showed that the production of reactive oxygen species contributes to autophagy and mitophagy and MFN2 can promote mitophagy through some specific mechanisms [31]. So we believe that autophagy and its relationship with the ROS/Mfn2 axis should be investigated in the future. Last but not least, mitochondrial biogenesis could also regulate mitochondrial dynamics. Some studies have demonstrated that DOX can lead to dysfunctional mitochondrial biogenesis by affecting the PGC-1α signaling pathway [32]. We speculate that mitochondrial biogenesis may also have a potential contribution in this process, and we believe it deserves further study in the future.

## Conclusion

In conclusion, we confirmed for the first time that the TSC-Exos could reduce mitochondrial fission and ROS production of cardiomyocytes by increasing the expression of Mfn2 to reduce Dox-induced cardiomyocyte apoptosis and improve cardiac function, which provides a potential new treatment scheme for the treatment of heart failure.

## Data Availability

The data that support the findings of this study are available from the corresponding author upon reasonable request.
